# Variation in condylar morphology in different malocclusion among Indians

**DOI:** 10.6026/973206300171134

**Published:** 2021-12-31

**Authors:** Swapna Sreenivasagan, Ashwin Mathew George, Sri Rengalakshmi

**Affiliations:** 1 Saveetha Dental College and Hospitals, Saveetha institute of medical and technical sciences (SIMATS), Saveetha University, Chennai-77 India

**Keywords:** Condyle, Temporomandibular joint, malocclusion, orthopantogram

## Abstract

Temporo-mandibular joint (TMJ) joint and the condyle of mandible are observed in the radiographs of the skull and the jaw. Therefore, it is of interest to assess the predictability of four different shapes of condyle in skeletal class I, II and III malocclusion. The four commonly visualized shapes are oval, bird beak, diamond and crooked were assessed using an ortho pantomogram (OPG). Each of the malocclusion was visualized for different shapes of the condyle. 987 OPGs were radiographically evaluated and the morphology of 1974 condylar heads was visualized. The shapes of the condyles were grouped under four different types. Data shows that oval shaped condyle was most common followed by bird beak. There was variability in the diamond and crooked shape and was lesser than the other types. Thus, the shapes of the condyle are useful predictable guide in deciding the nature of the occlusion.

## Background:

Temporomandibular joint (TMJ) is a ginglymoarthroidal joint that is formed by articulation between two bones, the condyle part of mandible and glenoid fossa at the base of the skull [1 - check with author 4]. The anatomic features of TMJ show variations among individuals and there are numerous factors that play a role in its shape and are concerned with the differences in functional loads imposed on the bone. There is a strong association between the form and function of the mandible and condyle among patients with different type of malocclusion [5]. Several studies have evaluated condylar circularity on symptomatic or asymptomatic samples in normal occlusion and malocclusion [6, [7 - check with author]. There are adequate data on how anatomical morphology or even the progression of symptoms [8]. Taking proper case history and diagnostic records are very essential to arrive at a proper diagnosis and formulate the treatment plan for the particular patient. Diagnostic aids used in orthodontic treatment planning were broadly divided into essential or non-essential diagnostic aids [9]. Management of TMD involves the work of dentists from various specialties. It is multifactorial in nature and requires proper examination and treatment planning. The first and foremost are the work of the specialist from oral medicine and radiology department to identify and diagnose the problem, followed by the work of the orthodontists, prosthodontists, or oral surgeons [2 - check with author]. In orthodontics, the position of the condyle may be of significance for two main purposes, either TMJ dysfunctions or to differentiate the body of mandible positions, which affect diagnosis and treatment[10]. Therefore, it is of interest to evaluate the morphology of the condyle in Angles Class I, Class II and Class III malocclusions using ortho pantomograms and to assess if the type of malocclusions is a confounding factor in the shape of the condyle.

## Materials & Methods:

The study consisted of evaluation of radiographs of 1974 condylar heads by examining condylar heads in a two- dimensional view on an ortho pantomogram. This study is done in an online setting of Dias software of the Saveetha Dental College. The sample was chosen between June 2019 to end of March 2020. Data collection was done using OPG of orthodontic patients and data to be verified by two examiners and records tabulated for verification by two examiners and the records tabulated for excel sheet. The independent variables of this study are the condylar morphology and the dependent ones are the various classifications of malocclusions.

## Results and Discussion:

**Figure 1:**
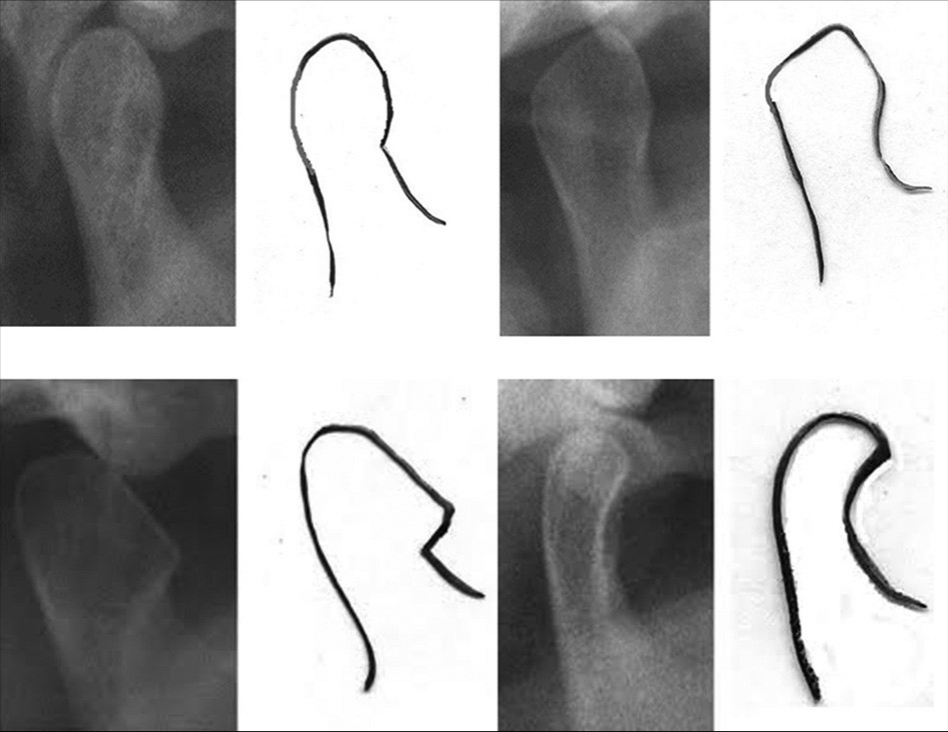
Shape of condyle on radiograph. The above figure represents the different shapes in condyle morphology; from top left clockwise the shape are oval, diamond, bird beak and crooked finger.

**Figure 2:**
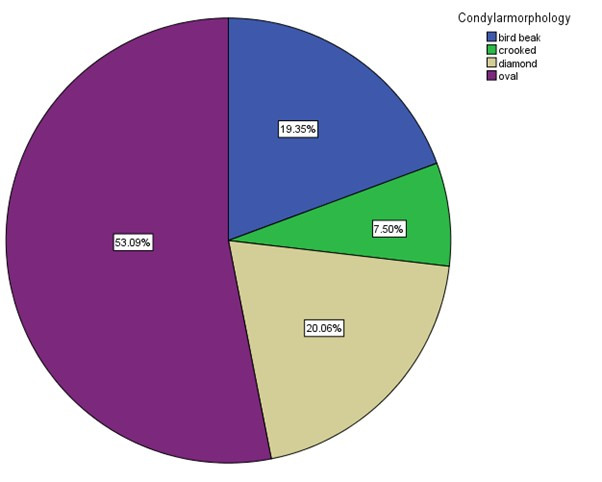
Pie chart depicting the frequency distribution of condylar morphology. The most common condylar morphology was oval 53.09% followed by diamond shape (20.06%), bird beak (19.35% and crooked (7.50%).

**Figure 3:**
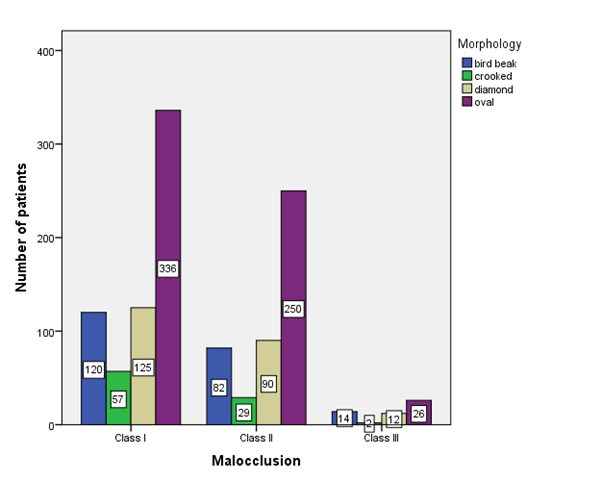
The bar graph depicts the association between the condylar morphology in Class I, II and III type of malocclusion. X-axis represents malocclusion and Y-axis represents the condylar morphology. Association between the different types of malocclusion and condylar morphology was done using chi square test and was not significant. The inference from this graph shows that in all the malocclusions the oval type of morphology is the most common. p value = 0.21 (>0.05%), statistically not significant.

The sample evaluated were 1974 condyles, which were studied from 987 cases with the age of subjects ranging from 16 to 45 years out of which 512 were female patients and 475 male patients (Figure 1). The types of mandibular condyles seen Indian population, the following shapes are witnessed in the Indian populations are oval, bird beak, diamond and crooked finger [1 - check with author]. The most common shapes are oval, bird beak, diamond followed by crooked in the order of accordance (Figure 2 and Figure 3).

Mandibular condylar shapes noted in both gender more in female cases and in both the gender more in female cases and in both the genders oval shaped in more common. The condyle is a site of facial growth and usually this growth is an upward and backward growth direction [11]. The position of the condyle is important where the practitioner plans to correct the occlusal plane, on modification on mandible growth and also in cases where the gonial angle of the mandible is altered. The shape of the mandible condyle varies in different stages of growth and among different subjects. In different imaging techniques using temporomandibular joint imaging orthopantomograms in remains of fundamental screening modality of TMJ abnormalities [12 - check with author]. Subjects with an increased mandibular plane angle are at a higher risk of fracture of the angle and can often present with its complications [13 - check with author]. Prediction of treatment based on anchorage preparation in skeletal malocclusion helps in efficient treatment results [14]. Orthodontists use various skeletal, dental, and soft-tissue analyses to diagnose and formulate a treatment plan [15].

The orthopantomogram can be used to visualize the maxillary and the mandibular arches, the dentition, the maxillary antrum, the nasal fossa, the temporomandibular joint, styloid process and the hyoid bone [16]. OPG is a part of an essential diagnostic aid as a part of radiographic examination in dentistry in diagnosing teeth, and the arches. It is a cost efficient and the effect dose of radiation is low [17]. Most common predictors include differences in maxillary mandibular morphology and its relationship [18]. Every individual will have some degree of asymmetry, as a completely symmetric face will not be esthetically pleasing, this minimal asymmetry often is unnoticed in the society [19].

According to this study, oval shaped was more common. In previous studies they have found round shape of the condyle in growing individuals in the anteroposterior slices [20], other studies have reported that rounded is most common at 66%, followed by flattened (17%) and angular forms (17%) . The anatomical morphology in the mandible starts getting established in early life and ossification of the bone even in the embryonic life, there is constant remodelling of the condylar bone and the morphology varies depending on the functional load and the activities of the jaw [21]. The prospect of investigation in this topic would be to determine if the different skeletal mal relations tend to generate different levels of functional load on the mandibular fossa [22]. There is often a finding of anterior condyle being displaced in the class I sample but the difference is less difference in the anterior and posterior article space of 1.3 and 1.7mm respectively.

There is a component of muscular overload onto the joint in Class II div 2 subjects and this is found to vary from other subjects who have different dentofacial morphologies. Thus orthodontic patients reporting with Class II div 2 malocclusion often present with different characteristic findings [23]. On clinical evaluation there is not always finding of centric condyles, mostly there are non-concentric condyles, this factor does not necessarily affect the TMJ signs and symptoms. A finding that is similar to other recorded literature is that there is a predilection towards non-concentric condyles with the anterior articular space reduced [24].

The low dose of radiation and being cost effective OPG is a preferred diagnostic aid. OPG is a readily available diagnostic aid to make observations. The most common morphology seen among various genders is oval. An efficient sample size collection based on power analysis along with parameters like the age, population would be an extensive knowledge in terms of diagnosis and classification [1 - check with author]. The variation and the pattern noticed among individuals in the morphological variations in the condyle and the fossa has now become an area of research [25]. The treatment in the field of orthodontics involves application of force on the teeth and often-orthopaedic force is directed towards the jaws in order to move the teeth or the jaws [26]. The direction of the orthodontic movement and the control of the tooth is of high significance to the orthodontist [13 - check with author,^27^]. The basis of orthodontic treatment is that when force is applied on to the teeth the alveolar bone adapts depending on the force applied [28].

In literature there are various studies that have assessed the various condylar morphology. There is difference in condyle morphology in various malocclusions. There are variations in male and female with men having a larger condyle than woman. The malocclusion that has a major change in the condyle is the transverse malocclusion. Another important finding Tadej et al had pointed out was the medio-lateral dimension as compared to anterior- posterior [29] . Seymour et al had evaluated the type of mandibular condyle had also pointed out that there is no difference in sex and in this sample he noted that 78.5% of mandible showed symmetry of condylar types . Ueda and his team had assessment on curvature analysis of the condyle and the study was done on a CT and he had found some gender related changes, of the patterns he noted that a bi-peak with a col profile was frequently in woman and bi-peak with a negative profile is more in male. Many other authors had described the condyle as round, point, angled, flat and irregular . An evaluation of condyle basic shape of the condyle on an opg but on a cone beam computed tomography the structure of the condyle can be examined better and the evaluation of the mandibular notch, coronoid and various configurations can be examined, as well as the aging often has a role on the condyle morphology . Many other Indian authors had also evaluated the morphology of condyle. In Sonal et al study they had found that the oval shape was common [1 - check with author]. There are often diseased conditions and disc displacements that can alter the shape and size of the condyle. The data was collected in the spreadsheet tabulated for each patient for the purpose of following the collected data, which included all the required personal details of the patient. The study evaluated 1974 condyles including light and left sides. The most common shape on the condylar head is the oval. Radiograph is only a two-dimensional view of a three-dimensional structure. The limitation of size of sample in all three malocclusion, various patients have undergone different treatment applications. The medical or symptomatic joint conditions are not known, as this is a retrospective study type. Growth status needs to be specified.

## Conclusion:

The predictability of condylar morphology is of significant importance. Data showed that all three types of skeletal malocclusion I, II and III, the oval shaped condyle was found to show the maximum occurrence. The next common shapes are diamond and bird beak and the crooked shaped condyle is the least common. There was no significant difference between the left and right side of the condyle. Results of the study suggest that reasonable predictability of condylar morphology with nature and any deviation from the oval shape would require the need for further investigation with relevance to any clinical significance.
